# The Dynamic Growth Exhibition and Accumulation of Cadmium of Pak Choi (*Brassica campestris* L. ssp. *chinensis*) Grown in Contaminated Soils

**DOI:** 10.3390/ijerph10115284

**Published:** 2013-10-25

**Authors:** Hung-Yu Lai, Bo-Ching Chen

**Affiliations:** Department of Post-Modern Agriculture, MingDao University, No. 369, Wenhua Rd., Peetow, Changhua County 52345, Taiwan

**Keywords:** bioconcentration factor (BCF), cadmium (Cd), dynamic accumulation, pak choi

## Abstract

The accumulation of heavy metals, especially cadmium (Cd), in leafy vegetables was compared with other vegetables. Pak choi (*Brassica campestris* L. ssp. *chinensis*) is a leafy vegetable consumed in Taiwan and its safety for consumption after growing in contaminated soils is a public concern. A pot experiment (50 days) was conducted to understand the dynamic accumulation of Cd by pak choi grown in artificially contaminated soils. The edible parts of pak choi were sampled and analyzed every 2–3 days. The dry weight (DW) of pak choi was an exponential function of leaf length, leaf width, and chlorophyll content. The accumulation of Cd increased when the soil Cd concentration was raised, but was kept at a constant level during different growth stages. Pak choi had a high bioconcentration factor (BCF = ratio of the concentration in the edible parts to that in the soils), at values of 3.5–4.0. The consumption of pak choi grown in soils contaminated at levels used in this study would result in the ingestion of impermissible amounts of Cd and could possibly have harmful effects on health.

## 1. Introduction

Soils contaminated with heavy metals (HMs) are major environmental problems throughout the world due to their adverse ecological effects. The remediation of HM-contaminated soils is a challenge because HMs do not degrade in the environment. Among the HMs, cadmium (Cd) contaminates soils primarily as a consequence of inappropriate disposal of industrial wastes, irrigation using contaminated water, application of fertilizers, or atmospheric deposition [1,2,3]. Cadmium is a non-essential element that has no metabolic use and is believed to cause damage even at low concentrations [4,5]. In plant tissues, excess Cd inhibits photosynthesis and respiration [6], and also reduces nutrient uptake [7]. The high mobility of Cd makes it easily taken up by crops, rapidly transferred to aerial parts, readily incorporated into the food chain, and consequently, a threat to human health [8,9]. 

Farmland contamination with Cd was reported in the last decade, and the public is now paying more attention to the potential toxicity of ingested crops [10,11]. In 1982, some farmlands in northern Taiwan were contaminated with Cd as a result of irrigation using the illegal effluent from a nearby chemical factory. Paddy rice grown in these soils accumulated high concentration of Cd in their grains [12]. The Soil and Groundwater Pollution Remediation Act (SGWPR Act) announced by Environmental Protection Administration (EPA) of Taiwan uses Soil Control Standards (SCSs) as the threshold for identifying contamination. Contaminated sites should conduct necessary remediation practices when their total concentrations (aqua regia soluble) of HMs are beyond those recommended in the SCSs. For farmlands, the SCS for Cd is 5.0 mg kg^−1^. However, some genotypes of paddy rice accumulated more than 0.4 mg Cd kg^−1^ (the regulative standard announced by Department of Health of Taiwan) in their grains even when the total Cd concentration in soils was less than the SCS [13]. 

Leafy vegetables accumulate higher concentrations of Cd than other crops [9,14,15]. It is predictable that leafy vegetables, like the popularly consumed pak choi, will accumulate harmful concentrations of Cd in the edible parts even when grown in soils containing low concentrations of Cd [16]. Many studies have been conducted to investigate the accumulation of Cd in harvest-ready leafy vegetables grown in soils with different Cd concentrations. To the author’s knowledge, however, no study has yet been conducted to examine the dynamic accumulation of Cd at different growth stages. If the dynamic accumulation of Cd is known, the harvest of vegetables grown in contaminated soils could be changed to an acceptable period that would minimize the amount of accumulated Cd in the harvested parts. The main objectives of this study are: (1) to understand the dynamic accumulation of Cd by pak choi, and (2) to assess the risk of uptake of Cd at different growth stages in pak choi grown in soils with different contamination levels of Cd.

## 2. Experimental Section

### 2.1. Soil Preparation

Soil samples were collected from the surface layer (0−30 cm) of an Inceptisol in central Taiwan. Soil samples were air dried, ground, and passed through 10, 80, or 100 mesh stainless steel sieves in accordance with different analyzing procedures. Basic soil characteristics of representative samples were then determined. Soil pH value (soil/water = 1/1; *w*/*v*) was determined with pH meter (Mettler Toledo EL20, Columbus, OH, US) using a glass electrode [17]. The electrical conductivity (EC) of saturation extract was determined according to [18]. Soil particle size distribution was measured by a hydrometer [19]. Water content and water-holding capacity (WHC) were determined by gravimetric method [20]. Organic carbon (OC) content of ground and sieved (80 mesh) soil samples was determined by the Walkley-Black wet combustion method [21]. 

Total concentration of Cd in ground and sieved (100 mesh) soil samples (3.0 g) was determined following digestion in 28 mL of aqua regia (7 mL HNO_3_ + 21 mL HCl). Digests were filtered through Whatman No. 42 filter papers, made up to a 100 mL volume, and analyzed for Cd content with an AAnlyst 200 flame atomic absorption spectrometer (FAAS, PerkinElmer, Waltham, MA, US). Suitable volumes of solutions of Cd(NO_3_)_2_·4H_2_O dissolved in deionized water (DI water) were sprayed on the sieved and air dried soil samples (5 mesh) to achieve their target Cd concentration (as an individual element) as: (1) Cd-CK: control with no Cd solution applied; (2) Cd-10: final total Cd concentration is 10 mg kg^−1^; and (3) Cd-20: final total Cd concentration is 20 mg kg^−1^. These artificially Cd-contaminated soils were subjected to three cycles of wet (50%−70% WHC)/dry (air-dried) incubation to simulate field conditions and to enable the added Cd to reach a steady state [22,23].

### 2.2. Pot Experiment

Air-dried and artificially Cd-contaminated soil samples (20.0 kg) were placed in rectangular pots (L 64 cm, W 48 cm, H 15 cm). Solutions of N (as urea), P (in KH_2_PO_4_), and K (in K_2_SO_4_) were added at 1.0 g N, 0.44 g P, and 1.0 g K pot^−1^ for all Cd treatments. A total of 130 pak choi (*Brassica campestris* L. ssp. *chinensis*) seeds were uniformly sown in each pot at about 1 cm depth from the soil surface. The pot experiment was conducted in a phytotron (25 °C, day/night = 12 h/12 h) for 50 days at MingDao University, with three replicates. Appropriate amounts of DI water were applied every 2−3 days to maintain soil moisture at 50%−70% WHC, which was determined by weighing the pot. 

The edible parts of pak choi were sampled randomly every 3−4 days from day 15th and 5−10 plants·pot^−1^ were harvested each time. The plant height (H), leaf length (L), and leaf width (W) of the largest expanded leaf was determined and recorded immediately after harvest. The chlorophyll content of each expanded leaf was determined by a chlorophyll meter (SPAD-502, Konica Minolta, Osaka, Japan); these measurements had been found to correlate closely with actual leaf chlorophyll content [24]. The fresh weight (FW) of each plant was also determined and recorded immediately after harvest. Dry weight (DW) was determined and recorded after rinsing each plant part with tap water, flushing with DI water, and then oven drying at 65 °C for 72 h.

### 2.3. Plant and Soil Analysis

Ground tissues (0.1−0.2 g) were digested by 10 mL of mixed concentrated acid (HNO_3_/HClO_4_ = 87/13 by volume). After filtering through Whatman No. 42 filter papers and made up to 25 mL, the digests were stored at 4 °C until further analysis. Soils were sampled and then air-dried and ground. Sieved (100 mesh) soil samples (3.0 g) were digested by aqua regia, made up to 25 mL, and filtered through Whatman No. 42 filter papers. The Cd concentrations in plant and soil digests were determined using a PerkinElmer AAnlyst 200 FAAS.

### 2.4. Quality Control and Statistical Analysis

Certified reference materials of soil (CRM 143 R) and standard reference materials of tomato leaves (SRM 1573a) were used to monitor the accuracy of data. The data were considered valid when the recovery rate (actual concentration/certified concentration) in the digestion processes was in the range of 90%−110%. Reagent blanks and three replicates of all samples were used to ensure accuracy and precision in the analysis. All of the quality control procedures conducted during the determination of Cd by FAAS followed NIEA M111.01C announced by Environmental Analysis Laboratory of Taiwan [25]. 

A statistical analysis was performed using SPSS (Statistical Package for Social Science, Armonk, NY, US). The differences in the growth rate and shoot Cd concentration among mean values of Cd treatments were evaluated by one-way analysis of variance (ANOVA) followed by LSD (least significant difference) test. Statistical significance was defined at the level of *p =* 0.05.

## 3. Results and Discussion

### 3.1. Basic Soil Characteristics

The texture of the soil used was sandy loam (sand 64%, silt 14%, clay 22%) with a moderate OC content (1.21%) and neutral acidity (pH 7.72). Its EC was 0.39 dS m^−1^ which is less than the threshold (4.0 dS m^−1^) of a saline soil. The initial total Cd concentration in soil was 0.8 ± 0.3 mg kg^−1^, which is less than the SCS of 5.0 mg Cd kg^−1^ imposed for farmlands in the SGWPR Act of Taiwan and can be regarded as a non-contaminated soil. After artificially spiking with Cd solutions, the final Cd concentration of Cd-10 and Cd-20 reached 10.5 ± 0.2 and 20.1 ± 0.8 mg kg^−1^, respectively, which were close to the target concentrations.

**Figure 1:**
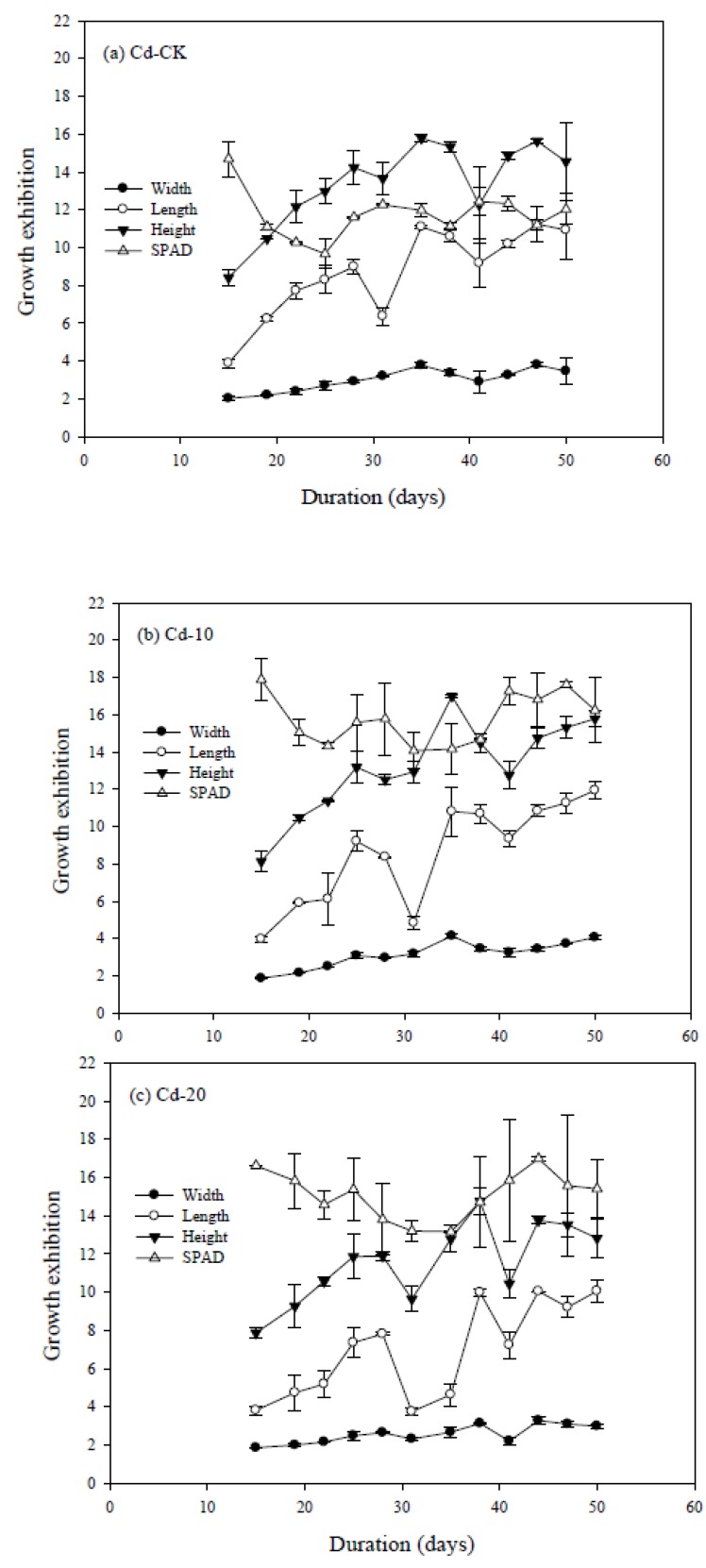
The dynamic exhibition of leaf length, leaf width, plant height, and chlorophyll content (SPAD) of pak choi grown in (**a**) Cd-CK; (**b**) Cd-10; and (**c**) Cd-20. Replicates: *n* = 3.

### 3.2. Dynamic Change of Physiological Characteristics

Figure 1 shows the dynamic exhibition of L, W, H, and SPAD for pak choi grown in artificially Cd-contaminated soils. In general, and except for SPAD, the L, W, and H increased with time. The L showed similar trends to H, although some of the values were evidently reduced. Since no unhealthy plants were observed during the pot experiment and the growth of plants is irreversible, the plant Cd content will not decrease once increased. For the three Cd treatments, some of the measurements seemed to be unreasonable, namely days 31, 35, and day 41. It is possible that these discrepancies resulted from sampling error, but it is difficult to explain the forgoing phenomena from the present data. 

An exponential equation (Equation (1)) was used to describe the dynamic change in DW of edible parts with the growing time and some of the unreasonable data were excluded:

DW_(t)_ = DW_0_ · e^g · t^ (1)

where DW_(t)_ is the DW (g plant^−1^) at a given time (days), DW_0_ is the initial DW, g is the growth rate (day^−1^), and t is a given time (days). 

**Figure 2:**
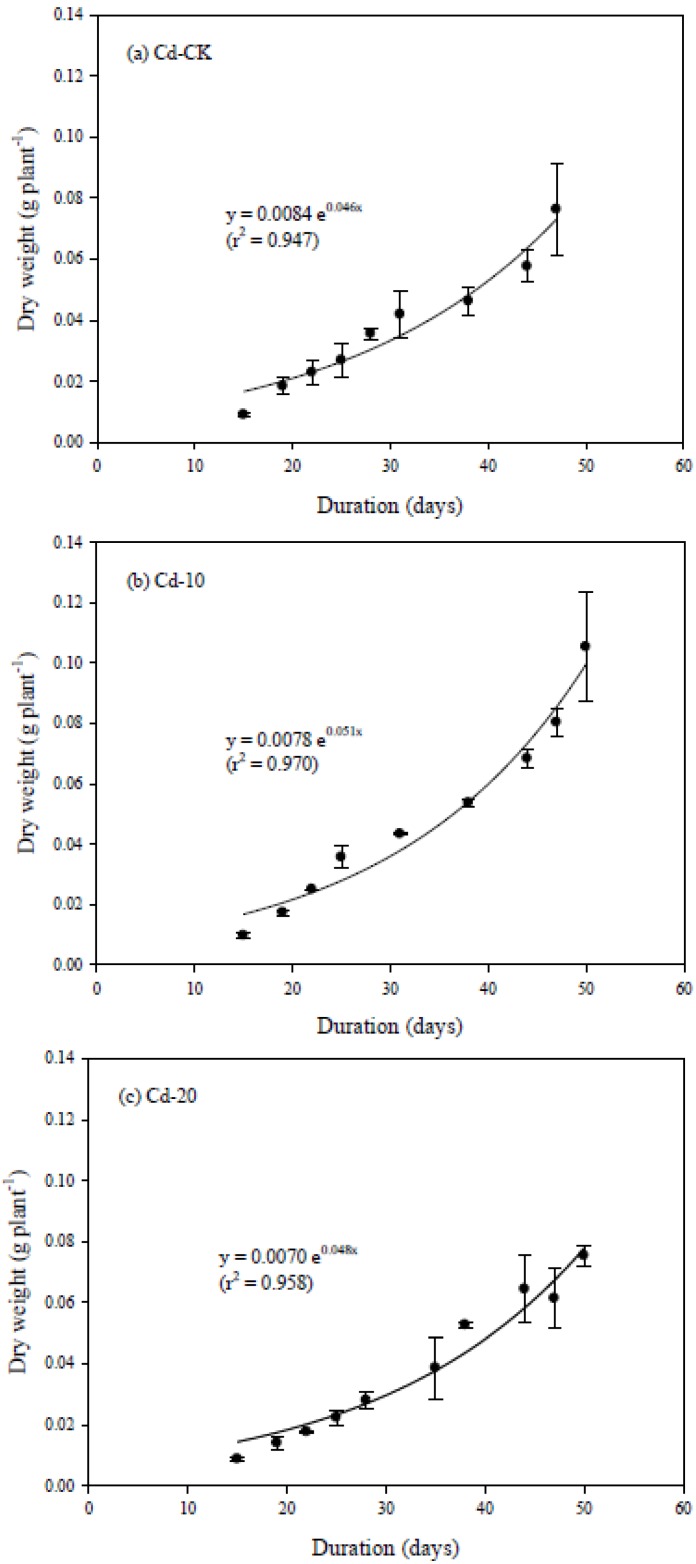
The dynamic change of dry weight of pak choi grown in (**a**) Cd-CK; (**b**) Cd-10; and (**c**) Cd-20. Replicates: *n* = 3.

Exponential equations well described the change in DW at different growth stages (Figure 2). For the three Cd treatments, the growth rate was at the level of 0.046 to 0.051 and had good correlation coefficients (r^2^ = 0.947 to 0.970). 

Even when growing in the Cd-treated soils, the growth rate for the Cd-10 and Cd-20 treatments showed a significant increase when compared to Cd-CK (*p* < 0.05), indicating that the growth of pak choi was not inhibited by the Cd concentration in soils. In agreement with a report by Chen *et al*. [16], the presence of Cd in soils promoted the growth of pak choi if the Cd concentration was below the toxic level.

### 3.3. Relationships between Physiological Characteristics

Nonlinear models using L, W, or SPAD were conducted by Cho *et al*. [26] to provide good representations of the leaf area (LA) and DW of cucumber. In this study, the physiological characteristics, namely L, W, and SPAD, of the largest leaves of pak choi grown in each treatment were determined during the pot experiment. A stepwise-multiple regression was conducted to describe the relationship between predicted DW (DW_predicted_) and the parameters determined, shown as Equation (2), with r^2^ of 0.893.

DW_predicted_ = 0.0282 × W + 0.00223 × L + 0.00235 × SPAD – 0.0894 (2)

DW_predicted_ = 0.893 × DW_determined_ + 0.0047 (3)

**Figure 3:**
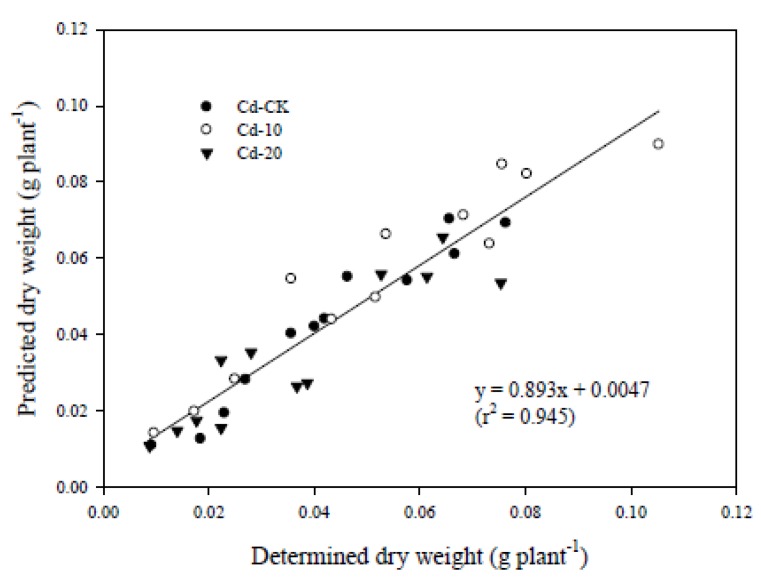
Linear relationship between the determined dry weight and the predicted dry weight of pak choi grown in various Cd-contaminated soils.

A significant linear relationship was found between the predicted DW and the determined DW (DW_determined_) and the r^2^ was 0.945 (Figure 3).

### 3.4. Bioconcentration and Dynamic Changes in Accumulated Cd Concentration

**Figure 4:**
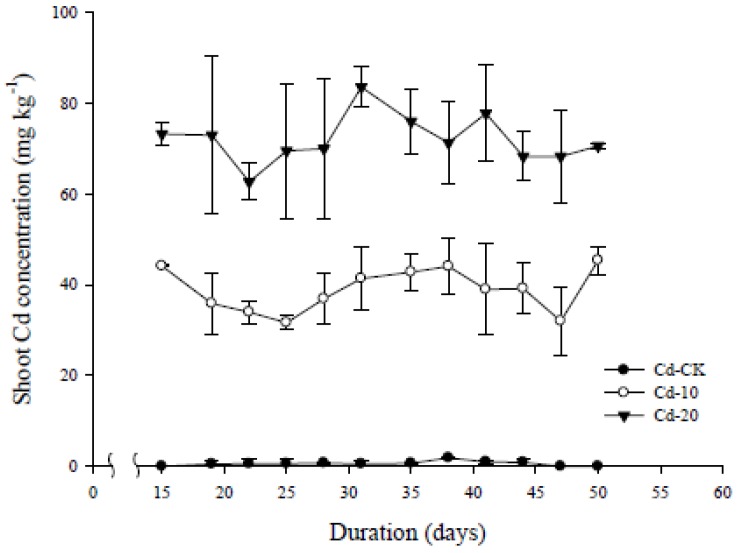
The dynamic change of Cd concentration (dry weight based) in the edible parts of pak choi grown in various Cd-contaminated soils. Replicates: *n* = 3.

Figure 4 shows the dynamic changes in Cd concentration (DW based) in the edible parts of pak choi grown in various Cd-contaminated soils. The accumulated Cd concentration significantly increased with increasing soil Cd concentration (*p* < 0.05). Although accumulated Cd concentrations changed at different growth stages, these changes were not drastic when the larger variations in some data were considered, and especially in Cd-20. 

The accumulated Cd concentrations during day 15 and day 50 were in the range of 31–46 mg kg^−1^ (average = 38.9 ± 4.8) and 62–84 mg kg^−1^ (average = 72.0 ± 5.3) for Cd-10 and Cd-20, respectively. Chen *et al*. [16] used artificially Cd-contaminated soils and planted pak choi at various Cd levels. The tissue Cd concentrations in pak choi grown in Cd-2 and Cd-10 were approximately 40 and 70 mg kg^−1^, respectively, which is in agreement with these previous experimental results. 

The dry weight based bioconcentration factor (BCF_DW_ = shoot Cd concentration/soil Cd concentration) was used to assess the accumulation capacity of pak choi. For Cd-10 and Cd-20, the BCF_DW_ reached 3.96 and 3.59, respectively (Table 1). In comparison with other experiments that studied pak choi in Cd-contaminated soils at similar concentrations, the BCF_DW_ reported in the present study was close to that reported by Chen *et al*. [16], but was approximately 1.7-fold higher than that reported by Chen *et al*. [23]. The phytoavailability of Cd in soil is primarily controlled by soil pH, redox potential, and cation exchange capacity (CEC) [27,28]. The mobility and phytoavailability of soil Cd increases when soil has a low pH value or CEC, which is mostly found in soils with a high sand content [29]. Compared with Chen *et al*. [23], the higher BCF_DW_ found in the present study and in Chen *et al*. [16] was possibly a result of the coarser texture (lower CEC) and lower pH (5.5).

**Table 1:** The dry weight based bioconcentration factor (BCF_DW_) of pak choi grown in various Cd-contaminated soils for 50 days.

Treatment	Soil Cd conc.	Shoot Cd conc.	BCF_DW_ *
	mg·kg^−^^1^		
Cd-CK	0.8 ± 0.3 ^#^	0.7 ± 0.5	0.80
Cd-10	10.5 ± 0.2	38.9 ± 0.8	3.96
Cd-20	20.1 ± 0.8	72.0 ± 0.3	3.59

^#^ mean ± standard deviation; Replicates (*n* = 3); * BCF_DW_ = shoot Cd concentration (dry weight based)/soil Cd concentration.

The continuous monitoring of yields during growth stages of different crops is difficult because destructive methods must be used. The development of non-destructive modeling can therefore be a very convenient and useful tool for the estimation of crop yields at various growth stages [26]. Similar to other regression models regarding L, W, LA, or SPAD for estimating crop production [23,30,31], a regression equation that used previous parameters was established in the present study to predict the DW of pak choi grown in Cd-contaminated soils. The modeled DW had a good linear relationship with the determined DW. This modeling (Equation (2)) can efficiently and accurately predict the DW at different growth stages without the need for destructive measurement. 

The SPAD has been used both to determine the chlorophyll concentration of leaves and also to estimate the nitrogen status and to guide fertilizer-N timing on corn and rice [32,33,34]. It is an effective tool for non-destructive estimation of total chlorophyll concentration and content across a range of plant ages, growing conditions, and genotypes [24]. However, successful use of the SPAD meter can be influenced by soil type, crop variety, growth stage, leaf position, and the sampling point on the leaf [32,35]. 

**Figure 5:**
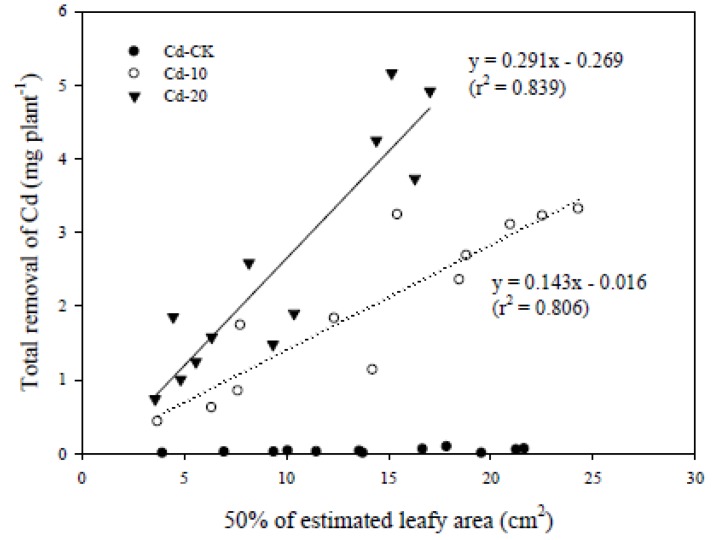
Linear relationship between 50% of estimated leafy area and the total removal of Cd by the edible parts of pak choi grown in various Cd-contaminated soils for 50 days. Replicates: *n* = 3.

The LA plays an important role in photosynthesis and crop yield. Although LA was not determined in the present study, it can be expressed as 50% of the product of L and W if the shape of leaf of pak choi is considered as a diamond. Good linear relationships were observed between 50% of estimated LA and the total removal of Cd by the edible parts of pak choi grown in Cd-10 (r^2^ = 0.806) and Cd-20 (r^2^ = 0.839) (Figure 5). When growing in Cd-contaminated soils, the total amount (mg plant^−^^1^) of Cd accumulated in the edible parts of pak choi increased because the estimated LA and DW increased, although the Cd concentrations did not change drastically. In other words, although the total mass of accumulated Cd increased with time, the increase in DW diluted the Cd concentration and thereby reduced the extent of the increase. However, it is difficult to distinguish between the effects resulting from dilution and from transpiration from the present data. 

**Figure 6:**
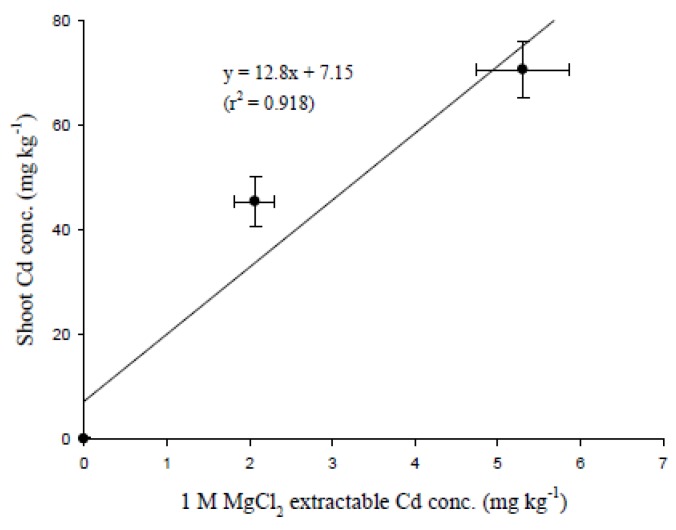
Linear relationship between 1 M MgCl_2_ extractable Cd concentrations in soils and that accumulated in the edible parts of pak choi grown in various Cd-contaminated soils for 50 days. Replicates: *n* = 3.

Once HMs enter the soil environment, they will react with soil particles and form different species, namely water soluble, exchangeable, or bound (to carbonates, organic matter, or oxides, *etc*.). In compliance with the strength of binding and phytoavailability, five fractions were separated by extracting with different chemical solutions [36]. The concentration of HM extracted by 1 M MgCl_2_ can represent the water soluble and exchangeable species, which should move with the soil solution. Because approximately 90% of the water taken up by plants will be lost through transpiration, LA dictates the amounts of water that pass through plant tissues and are lost from the stomata. During transpiration, the water soluble or exchangeable fractions of soil Cd will transfer together with water within plant tissues. The accumulation of Cd in plants is mainly driven by transpiration, which depends on LA, and higher transpiration generally leads to higher shoot Cd levels [37]. Plants thus accumulate more HM in their tissues if the transpiration rate is high or if most of the Cd in soils is primarily present as water soluble or exchangeable fractions. Soil samples from the pot experiment in the present study showed that approximately 19% to 27% of the total Cd in the soils could be extracted with 1 M MgCl_2_. There was a good linear relationship between this extractable Cd in the soil and that accumulated in the edible parts of pak choi (r^2^ = 0.918) (Figure 6). However, although the extractable soil Cd seems to be available for uptake into pak choi, further studies will be needed since only three Cd treatments were used in the present study. 

**Figure 7:**
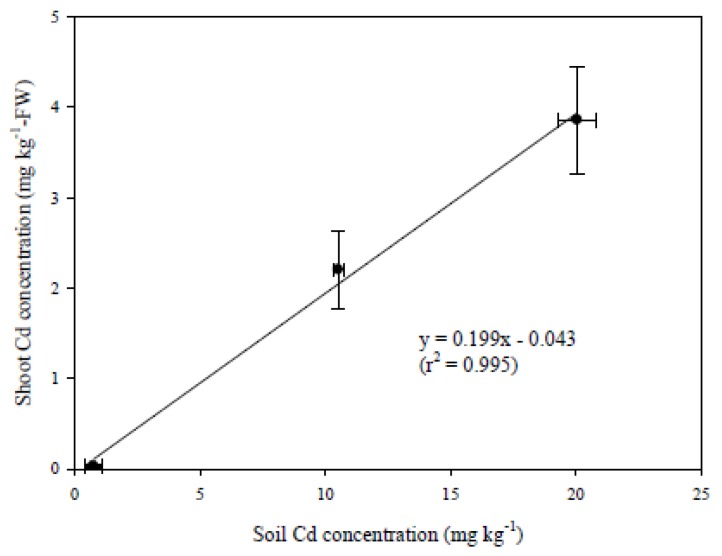
Linear relationship between Cd concentrations in soils and that in the edible parts of pak choi grown in various Cd-contaminated soils for 50 days.

Regression of the FW based average Cd concentrations in the edible parts of pak choi grown in Cd-CK, Cd-10, and Cd-20 with soil Cd concentrations yielded a good linear relationship between Cd concentration in soil (Cd_soil_) and the FW based Cd concentration in the edible parts (Cd_shoot_) (Figure 7). The BCF_FW_ (slope of the linear regression) was 0.199, indicating that pak choi will accumulate 0.199-fold of the Cd concentration (FW based) in the soil in its edible parts when growing in artificially Cd-contaminated soils. In Taiwan, the SGWPR Act was announced in 2000 and SCS was used as a threshold for determining soil pollution. The SCS is 5.0 mg kg^−1^ for farmlands and 20 mg kg^−1^ for non-farmlands. Approximately 0.952 mg kg^−1^ (FW based) of Cd will accumulate in the edible parts of pak choi grown in soil contaminated with 5.0 mg Cd kg^−1^ according to the BCF_FW_ obtained in this study. Based on the daily consumption of vegetables suggested by Department of Health of Taiwan and if 300 g (FW based) of pak choi is consumed, the daily uptake of Cd for each person will reach 286 μg. This value is far beyond the provisional tolerable weekly intake (PTWI) (equal to 60 μg day^−1^ for an average adult weighing 60 kg) suggested by Joint FAO-WHO Expert Committee on Food Additives [38]. The European Union maximum permitted concentrations in foodstuffs for leafy vegetables is 0.2 mg kg^−1^ (FW based). Using this BCF_FW_ for pak choi, this vegetable will accumulate more than 0.2 mg Cd kg^−1^ (FW based) if the soil Cd concentration reaches 1.23 mg kg^−1^. Therefore, pak choi should not be planted in Cd-contaminated soils where Cd is present at the concentrations used in this study because of the possible harmful effects due to consumption based on the available literature. However, aging of contaminated soil decreases HM phytoavailability [39,40]. The accumulation of Cd might be lower if pak choi is grown in soils where Cd contamination took place years earlier, unlike the artificially contaminated soils used in the present study. In addition, even after oral intake of pak choi, not all of the Cd contained within the edible parts will be trophically available because of subcellular compartmentalization [41]. 

For the pak choi used in the present pot experiments, no drastic change was noted for the concentration of Cd in the edible parts regardless of soil concentration (Figure 4). The accumulated Cd concentration was only related to the Cd concentration in soil if the larger outliers in the data were disregarded. Oral intake of HMs from soil medium can be calculated by Equation (4) [42]:

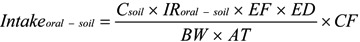
(4)

Intake_oral-soil_: exposure dose of oral intake (mg kg^−1^ day^−1^)
C_soil_: concentration of concerned pollutant (mg kg^−1^)
IR_oral-soil_: ingestion rate (mg day^−1^)
EF: exposure frequency (day year^−1^)
ED: exposure period (year^−1^)
BW: body weight (kg)
AT: average time (day)
CF: conversion factor (kg mg^−1^)

After determining the L, W, and SPAD of the largest leaf, one can predicted the DW of pak choi using Equation (2). If an allowable Intake_oral-soil_ is taken into account, the IR_oral-soil_ can be obtained for a given EF, ED, BW, and AT. Lai *et al*. [43] reported that the extension of growth period promoted the accumulation of Cd of different garden flowers. In Taiwan, a typical growing period for pak choi is 30 to 60 days. However, harvesting pak choi at early growing stages will not decrease the accumulated Cd concentration in the edible parts. 

## 4. Conclusions

Experimental results of the presented study show that pak choi is a Cd accumulator which accumulated 3.5–4.0 times more Cd concentration than the soil concentration. Once it was grown in the Cd-contaminated soils, the Cd concentrations in the edible parts kept at a constant level during different growth stages. Both leaf area and soil Cd concentrations play important roles regarding the accumulation of Cd of pak choi. 
